# Prospective study of the effect of rituximab on kidney function in membranous nephropathy

**DOI:** 10.1093/ckj/sfae179

**Published:** 2024-06-18

**Authors:** Durga A K Kanigicherla, Angie A Kehagia, Babak Jamshidi, Lina Manounah, Anna Barnes, Hannah Patrick, Helen Powell, Catrin Austin, Stephen Norton, Lisa Willcocks, Megan Griffith, Fiona Braddon, Retha Steenkamp, William S McKane, Arif Khwaja

**Affiliations:** Manchester Institute of Nephrology and Transplantation, Manchester, UK; King's College Technology Evaluation Centre (KiTEC), UK; King's College Technology Evaluation Centre (KiTEC), UK; King's College Technology Evaluation Centre (KiTEC), UK; King's College Technology Evaluation Centre (KiTEC), UK; National Institute for Health and Care Excellence, UK; National Institute for Health and Care Excellence, UK; National Institute for Health and Care Excellence, UK; National Institute for Health and Care Excellence, UK; Cambridge University Hospital NHS Trust, UK; Imperial College Healthcare NHS Trust Renal Unit, UK; UK Kidney Association & UK National Registry of Rare Kidney Diseases, UK; UK Kidney Association & UK National Registry of Rare Kidney Diseases, UK; Sheffield Kidney Institute, UK; Sheffield Kidney Institute, UK

**Keywords:** eGFR, membranous nephropathy, remission, rituximab

## Abstract

**Background:**

Patients with membranous nephropathy (MN) and poor kidney function or active disease despite previous immunosuppression are underrepresented in clinical trials. It is unknown how effective rituximab is in this population.

**Methods:**

This prospective, multi-centre, single-arm, real-world study of patients with active MN [urine protein-creatinine ratio (uPCR) >350 mg/mmol and serum albumin <30 g/L, or a fall in estimated glomerular filtration rate (eGFR) of at least 20% or more over at least 3 months] evaluated rituximab in those with contraindications to calcineurin inhibitors and cytotoxic therapy. The primary outcome was change in rate of eGFR decline before and after rituximab. Complete or partial remission were defined as uPCR <30 mg/mmol or uPCR <350 mg/mmol with a ≥50% fall from baseline, respectively.

**Results:**

A total of 180 patients [median age 59 years, interquartile range (IQR) 48–68] received rituximab and were followed up for a median duration of 17 months. Seventy-seven percent had prior immunosuppression. Median eGFR and uPCR at baseline were 49.2 mL/min/1.73 m^2^ (IQR 34.4–80.6) and 766 mg/mmol (IQR 487–1057), respectively. The annual rate of decline of eGFR fell from 13.9 to 1.7 mL/min/1.73 m^2^/year following rituximab (Z score = 2.48, *P *< .0066). At 18 months 12% and 42% of patients were in complete or partial remission, respectively. Rituximab was well tolerated; patient survival was 95.6% at 2 years and in patients in whom eGFR was available, kidney survival was 93% at 2 years.

**Conclusion:**

Rituximab significantly reduced the rate of eGFR decline in active MN including those who had received prior immunosuppression or with poor baseline kidney function.

KEY LEARNING POINTS **What was known:**

- Patients with membranous nephropathy (MN) and poor kidney function or active disease, despite previous immunosuppression, are underrepresented in clinical trials.
- The effectiveness of rituximab in this specific MN patient population was unknown due to their exclusion from most clinical studies.
- Current treatment options like calcineurin inhibitors and cytotoxic therapy have limitations, including nephrotoxicity and significant side effects.

**This study adds:**

- Rituximab significantly reduced the rate of kidney function decline in patients with active MN, including those with prior immunosuppression or poor baseline kidney function.
- The study demonstrated both complete and partial remission in a considerable percentage of patients, altering the course of MN in a challenging patient group.
- The findings highlight rituximab's tolerability and positive effects on kidney survival, expanding its use as first-line therapy for MN with active disease.

**Potential impact:**

- This study could influence day-to-day medical practice by introducing rituximab as a viable treatment option for MN patients with prior immunosuppression or poor kidney function.
- The findings could lead to a shift in treatment guidelines and policies, considering rituximab for broader use in challenging MN cases.
- The study's conclusions about the safety and efficacy of rituximab may enhance patient outcomes by slowing the advancement of end-stage renal disease, but it also emphasizes the need for methods to maximize remission rates in a greater number of patients.

## INTRODUCTION

Membranous nephropathy (MN) is the most common cause of primary nephrotic syndrome in adults, with an estimated incidence of around 12 per million per year [1]. Thirty percent of patients undergo spontaneous remission, whilst 40%–50% of those with ongoing nephrotic syndrome develop end-stage kidney failure over a period of 10 years [2]. Whilst several target antigens have been identified in the pathogenesis of MN [3], autoantibodies to the phospholipase A_2_ receptor (PLA2R) are present in 70% of patients and are a highly sensitive biomarker of disease activity [4].

Clinical guidelines suggest using supportive therapy followed by immunosuppression for those with persistent nephrotic syndrome [5]. Calcineurin inhibitors (CNIs) such as ciclosporine [6] or tacrolimus [7] are used widely but are nephrotoxic, are associated with a high relapse rate on discontinuation and are not effective in patients with progressive MN [8]. Cytotoxic therapy with alkylating agents such as cyclophosphamide combined with glucocorticoids have a potent, B-cell-depleting effect, reduce the production of autoantibodies and are effective in 60%–83% of patients [9–11]. However, such therapy has significant toxicity including myelosuppression, infertility, and increased risk of cancer, diabetes, hypertension, osteoporosis and mood disturbance [8]. Rituximab is a well-tolerated, selective B-cell-depleting monoclonal antibody that is superior to both supportive care and ciclosporin in inducing remission [12–14]. Its use was first reported in a series of eight patients in 2002 [15]. More recently, it has been shown to be equivalent to cyclophosphamide, although in an underpowered study [16], and low-dose rituximab combined with tacrolimus induced remission in fewer MN patients than a combined corticosteroid–cyclophosphamide regimen [9]. A key limitation of these trials is that they were mostly in patients with well-preserved kidney function who did not receive prior immunosuppression.

As a previous randomized control trial (RCT) in MN took 10 years to recruit 108 patients [8], National Health Service England (NHSE) initiated a Commissioning through Evaluation (CtE) scheme to rapidly develop an evidence-based treatment policy for rituximab in MN using prospective data collected by the UK National Registry of Rare Kidney Diseases (RaDaR), Hospital Episodes Statistics (HES) and Office of National Statistics (ONS) mortality data. This study was not an RCT but a prospective, national audit, with the aim of using data linkage to collect real-world data on the efficacy of rituximab in preventing decline in kidney function in a population with MN who had largely been excluded from RCTs.

## MATERIALS AND METHODS

### Design and patient population

We used a prospective, multi-centre, registry-based, time-limited design to determine the safety and effectiveness of rituximab for the treatment of MN. The primary outcome was rate of change of estimated glomerular filtration rate (eGFR) pre- and post-rituximab. Secondary outcomes included complete and partial remission rates. There was no control arm as patients had absolute or relative contraindications to all other immunosuppressive therapy. Therefore, a within-patient, before and after treatment analysis was undertaken.

MN was diagnosed by renal biopsy or presence of anti-PLA2Rab with proteinuric disease. Patients from 52 NHS adult renal centres in England were eligible for rituximab if secondary causes of disease were excluded and they had evidence of ongoing active disease defined as:

- urine protein:creatinine ratio (uPCR) >350 mg/mmol AND
- serum albumin (SA) <30 g/L, OR a fall in eGFR ≥20% over 3 months OR longer, based on at least three measurements.

Patients had to be on maximum tolerated renin–angiotensin–aldosterone system (RAAS) blockade and to have had failure, intolerance or contraindication to conventional immunosuppression with CNIs and cytotoxic therapy (combination of alkylating agents and steroids). Patients unable to tolerate RAS blockade were also not excluded from the study. Failure of therapy was defined as evidence of ongoing active disease at least 3 months after completion of treatment with CNIs or cytotoxic therapy. Intolerance to cytotoxic therapy included threatened fertility, previous urothelial cancer, previous hospitalization with infectious complications or previous bone marrow suppression or hepatitis. For CNIs, intolerance was defined as fall in eGFR of at least 20% or an eGFR <60 mL/min/1.73 m^2^ or previous hospitalization with metabolic or infectious complications of CNIs. For steroids, intolerance was defined as diabetes or risk factors for steroid-induced diabetes, osteopenia or osteoporosis and a history of mood disturbance on steroids. Patients unable to comply with the monitoring requirements of CNIs or cytotoxic therapy were also eligible for rituximab.

Patients were scheduled to receive rituximab in two doses of 1000 mg intravenously, 14 days apart, with follow-up at 3, 6, 9, 12, 18 and 24 months to monitor renal parameters, anti-PLA2Rabs and complete a survey on health-related quality of life (HRQoL). Patients could be re-treated for relapse with rituximab if they had sustained partial remission for longer than 6 months after previous dosing. Use of *Pneumocystis* prophylaxis was left to the discretion of the treating clinician as per local guidance.

This pragmatic study allowed for recruitment and follow-up of 180 patients during a 3-year window. Patients with recurrent MN post transplantation are not included in this analysis. The study aimed to analyse all the data available within 3 years from the recruitment of the first patient. As a result, there were varying degrees of follow-up for the patients in the study.

### Procedures

The data were anonymized by RaDaR and a study subject ID was shared with NHS-Digital and ONS. Individual patient records were linked to routinely collected HES and ONS mortality records, as well as the renal datasets from the follow-up visits.

### Statistical analyses

The outcomes following rituximab were analysed using eGFR (Chronic Kidney Disease Epidemiology Collaboration formula [5]), uPCR, SA and anti-PLA2Rab levels before and after treatment. To assess the effect of rituximab on eGFR change, a mixed model linear regression was performed to identify which variables, including rituximab, age, sex, uPCR, SA, anti-PLA2Rab levels, prior immunosuppression, other supportive treatments and diabetes, predicted the rate of standardized eGFR decline. Historical eGFR and SA measurements prior to rituximab allowed the rate of change over time to be compared at paired equal time intervals (12, 18 or 24 months) before and after its administration using paired t-tests. Insufficient historical data points precluded reliable curve fitting for uPCR, so the effect of rituximab was addressed in a paired t-test between 0 and 24 months. The rate of eGFR decline before and after rituximab was also compared by Mann–Whitney U test and paired t-tests as appropriate. The rate of eGFR change for each case was defined as eGFR change divided by the temporal distance (in months) between baseline and most distal recorded time for each period (pre and post). End-stage kidney disease (ESKD) was defined as the need for dialysis or transplantation.

Complete remission was defined as uPCR <30 mg/mmol and partial remission as an improvement in uPCR <350 mg/mmol and at least a 50% fall from baseline. Regression models at 12 and 24 months were built to analyse the relationship between remission status and clinically relevant predictors including age, sex, prior immunosuppression, other supportive treatments, comorbidities, previous medical conditions and duration of MN, baseline renal and anti-PLA2Rab parameters. Multivariable Cox regression models were run for time to remission. HRQoL impact was determined by change over time of the EQ-5D-5L (European Quality of Life 5 Dimensions 5 Level Version) score [17].

Adverse reactions, adverse events resulting in hospitalizations exceeding 24 h and deaths during follow-up until last censoring post-treatment were analysed by primary diagnosis code and chapter of the International Classification of Diseases, Tenth Revision (ICD-10) code. People still alive at the final follow-up assessment were censored at that date in the survival analysis. ONS death data were used to determine the date of death. Factors determining hazard rate for death were examined by Cox regression.

### Ethical approval

RaDaR holds the Research Ethics Committee (REC) approval for patient data to be collected as part of the scheme. For data collection and analysis by KiTEC, ethical approval was granted by South West—Central Bristol REC (ref: 14/SW/1088; IRAS: 239 534). The protocol, patient consent and patient information sheet are in the Supplementary data.

## RESULTS

### Patient characteristics

A total of 180 patients were recruited from 38 participating centres with the first patient recruited in August 2018 and last patient in January 2020. Baseline demographic and clinical characteristics are presented in Table 1 (S1  S2 see Supplementary data, S1 and S2 for comorbidities and treatment characteristics). Sixty-seven percent of subjects were men and the median age was 59 years [interquartile range (IQR) 48–68]. Of note, 77% of patients had received prior immunosuppression and the median eGFR at baseline was 49.2 mL/min/1.73 m^2^ (IQR 34.4–80.6). In the 153 patients in whom anti-PLA2Rab levels were available, 75% were positive. Eighty-eight percent of patients were on RAAS blockers. All patients received the first dose of rituximab; two patients did not receive a second dose and were excluded from the analysis. Overall, 178 received the second dose on average 13 days (standard deviation 7.15 days) later. Thirty-four patients received three or more doses during follow-up. Median follow-up was 17 months.

**Table 1:** Baseline characteristics.

Characteristic	Results	Number of patients
Years of MN disease	1.78 (0.7–6.2)	180
Number with biopsy-proven disease, *n* (%)	165 (92)	180
Demographics		
Age (years)	59 (48–68)	180
Sex (males), *n* (%)	120 (67)	180
Weight (kg)	84.6 (72.3–99)	179
Height (cm)	171.8 (165–177.8)	176
BMI (kg/m^2^)	29 (24.8–32.9)	175
Ethnicity		180
Asian	31	
Black	17	
Chinese	2	
White	119	
Other mixed/ethnic	7	
Not recorded	4	
Clinical parameters		
Previous immunosuppression, *n* (%)	138 (77)	180
RAAS blockade, *n* (%)	159 (88)	180
Systolic blood pressure (mmHg)	137 (124–154)	180
Diastolic blood pressure (mmHg)	80 (73–89)	180
uPCR (mg/mmol)	766 (487–1057)	177^a^
eGFR (mL/min/1.73 m^2^)	49.2 (34.4–80.6)	180
SA (g/L)	26.5 (22–31)	180
Positive for anti-PLA2R (titre level >14 RU/mL), *n* (%)	114 (75)	153^b^
Anti-PLA2R titre if positive for anti-PLA2R (RU/mL)	97 (47.5–198)	114
HRQoL^c^	0.85 (0.17)	175

Data are median (IQR) or *n* (%).

a uPCR at baseline was not available for three people.

b Results from one site (*n* = 11) were excluded due to a different assay being used. Data were missing or incorrectly entered for a further 16 patients.

c For HRQoL, mean (standard deviation) is shown.

BMI, body mass index.

### Kidney function, proteinuria and SA

**Figure 1:**
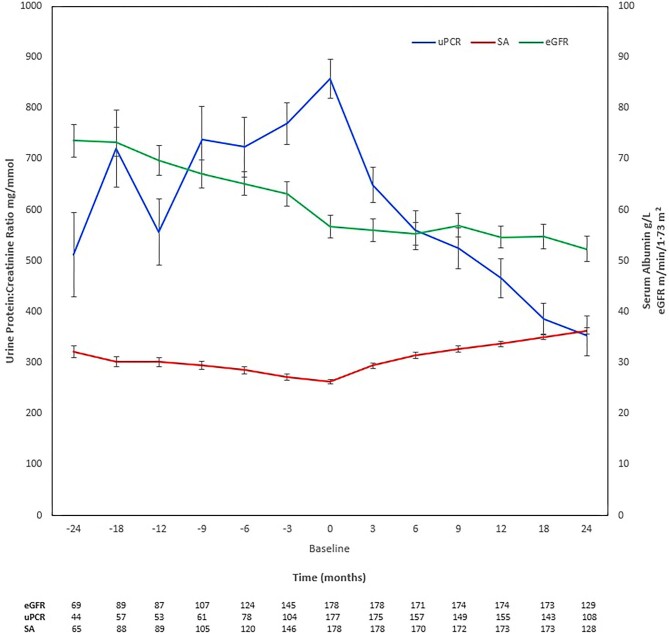
Change in eGFR, uPCR and SA before and after rituximab (first dose given at 0 months). Data points show means, and standard error bars show 95% CI. The numbers of patients for whom readings were taken at each timepoint are presented below the graph; variation in numbers was due to dropout and missing readings.

eGFR data were available for 173 patients at 18 months and 129 patients at 24 months. Changes in eGFR, uPCR and SA are presented in Fig. 1. Patient survival at 2 years was 95.6% and in those where eGFR was available at 2 years, kidney survival was 93% (Supplementary data, S7). There was a significant reduction in the rate of eGFR decline, with a clear reduction in uPCR and increase in SA after rituximab. Rituximab lowered the annual rate of eGFR decline from 13.9 to 1.7 mL/min/1.73 m^2^/year (number of timepoints before and after = 6, Z score = 2.48, *P *< .0066). The median eGFR at 24 months pre-infusion was 69.2 mL/min/1.73 m^2^ (IQR 53.6–96.2), which dropped to 49.1 mL/min/1.73 m^2^ (IQR 34.4–80.2) at baseline (when rituximab was given) and plateaued at 47.2 mL/min/1.73 m^2^ (IQR 27.6–75.5) by 24 months. When contrasting pairs of eGFR measures at 12, 18 and 24 months pre- and post-rituximab, each revealed a demonstrable trend change at 12 (t = 4.4, df = 80, *P *< .0001), 18 (t = 4.12, df = 80, *P *< .0001) and 24 months (t = 2.9, df = 46, *P *= .0029). There was a similar, significant increase in SA after rituximab at 12 months (t = 6.38, df = 82, *P *< .0001), 18 months (t = 6.66, df = 79, *P *< .0001) and 24 months (t = 4.98, df = 41, *P *< .0001) paired timepoints. uPCR also decreased over time from a median of 772 mg/mmol (IQR 490–1061) at baseline to 197 mg/mmol (IQR 95–487) at 24 months (t = 9.61, df = 106, *P *< .0001). In the 34 patients with an eGFR <30 mL/min/1.73 m^2^ at baseline, 10 (29%) achieved complete or partial remission at 18 and 24 months but 7 (21%) required renal replacement therapy during the follow-up period.

### Predictors of eGFR decline

Mixed model regression analysis revealed that rituximab predicted a lower rate of eGFR decline (t = –3.22, df = 77.17, *P *= .0019). Diabetes (t = 2.57, df = 60.85, *P *= .013) and baseline uPCR (t = 2.06, df = 97.7, *P *= .042) predicted a steeper rate of eGFR decline. There were no other predictors, including baseline eGFR or anti-PLA2Rab titres or previous immunosuppressive therapy. These results did not change after excluding nine patients who subsequently received additional immunosuppression (CNIs and alkylating agents) after rituximab in a sensitivity analysis.

### Remission and predictors of remission

**Figure 2:**
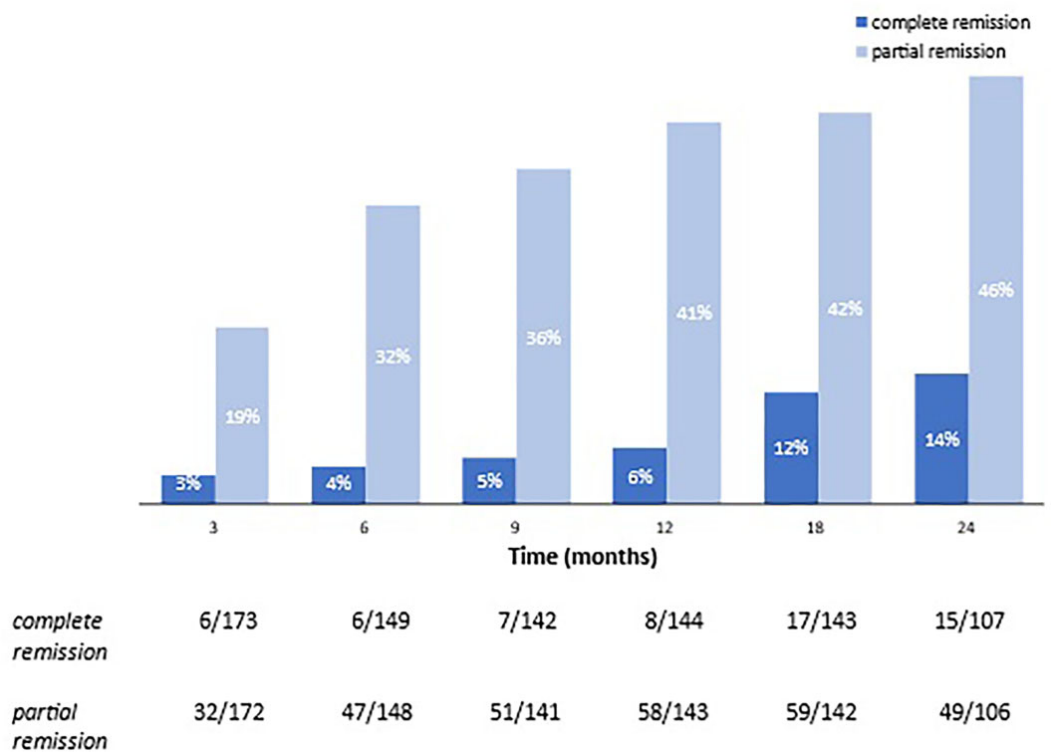
Proportion of patients reaching complete or partial remission at each follow-up timepoint after a cycle of rituximab. Data presented below the graph are the number of cases/total sample size at each follow-up timepoint, which differs between partial and complete remission, as the latter does not depend on baseline uPCR. Variation in numbers is due to dropout and missing readings.

For the 143 patients in whom uPCR data were available at 18 months, 17 (12%) were in complete remission and 59 (42%) were in partial remission. For the 107 patients with available data at 24 months, 15 (14%) and 49 (46%) were in complete and partial remission, respectively (Fig. 2). During the fixed 3-year window for recruitment and follow-up, 129 patients completed follow-up to 24 months. Of those who had uPCR data available, 15/107 patients were in complete remission (14%) and 49/106 were in partial remission (46%) at 24 months (Fig. 2). In the remaining 43 patients who did not achieve either complete or partial remission, uPCR still fell by 37% and 29% at 18 and 24 months, respectively.

Women were likely to achieve complete remission faster than men [hazard ratio (HR) 2.66, 95% confidence interval (CI) 1.54–4.59] and diabetes predicted longer time to remission (both complete and partial) (HR 0.39, 95% CI 0.3–0.5). Baseline proteinuria was the only consistent predictor of complete and partial remission at 12 and 24 months (Beta = –0.002, odd ratios 0.99, 95% CI 0.997–0.999), but the significance of this is questionable given that remission is a function of uPCR.

### Immunological remission and proteinuria

**Figure 3:**
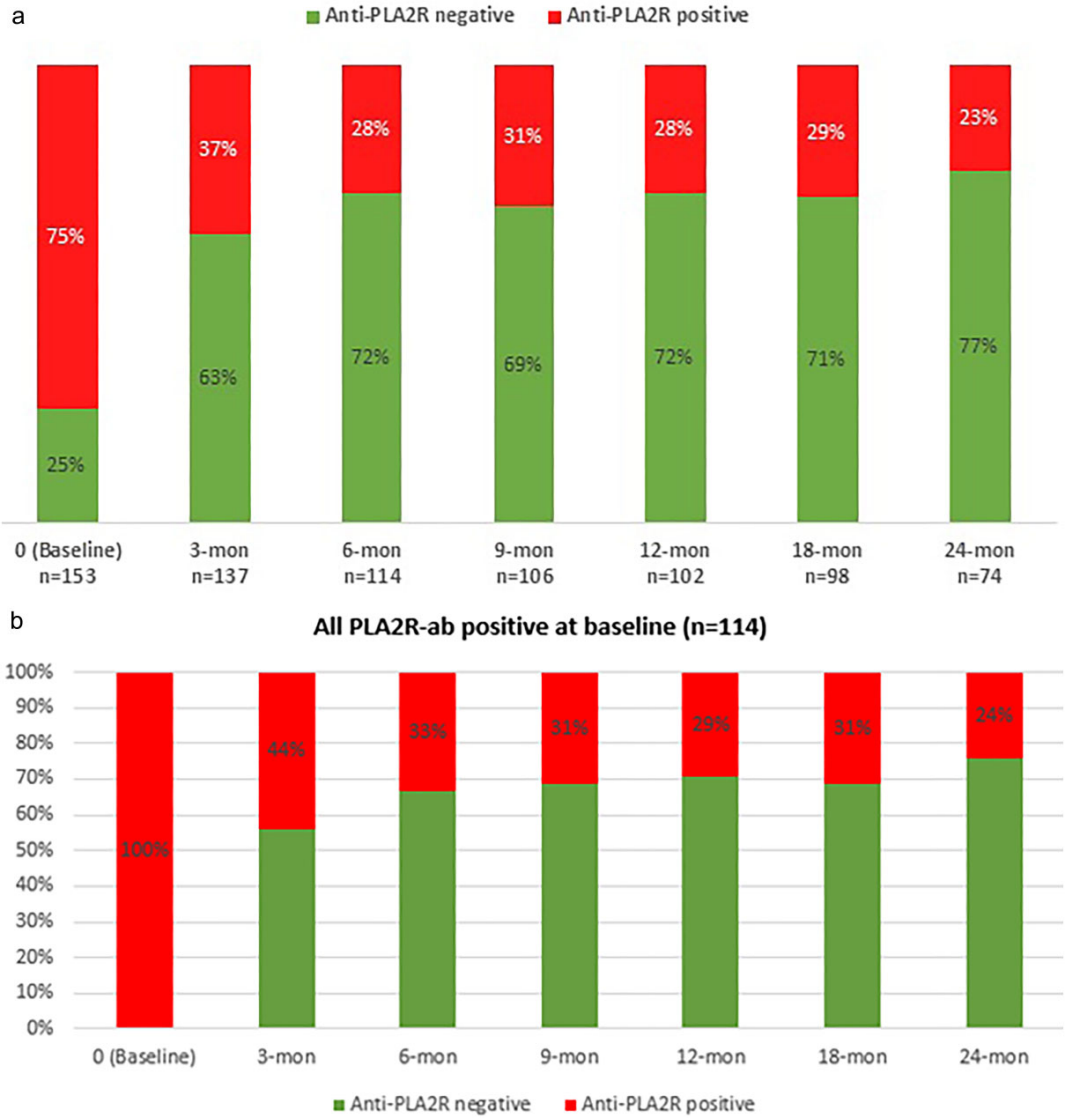
(**a**) Proportion of patients within the cohort who tested positive for anti-PLA2Rab after rituximab (includes all patients where tested). Proportion (%) of anti-PLA2Rab-positive and -negative patients at baseline (0 months) and up to 24 months following rituximab. The numbers of patients for whom readings were taken at each timepoint are presented; variation in numbers was due to dropout and missing readings. (**b**) Proportion of patients within the cohort who tested positive for anti-PLA2Rab at baseline and serial positivity after rituximab.

Rituximab significantly reduced anti-PLA2Rab levels (Fig. 3a and b), and more patients in immunological remission achieved either complete or partial clinical remission compared with those who remained anti-PLA2Rab positive at 12 months [Χ^2^ (1, *N* = 102) = 10.99, *P *< .00092] and 24 months [Χ^2^ (1, *N* = 74) = 10.38, *P *< .0013]. At baseline, 75% of subjects were anti-PLA2Rab positive, which declined to 28% at 12 months and 23% at 24 months. Nine of 27 (33%) patients with baseline anti-PLA2Rab titres <50 relative units (RU)/mL achieved clinical complete or partial remission at 18 months (Supplementary data, S3a). 15/36 (42%) of patients with baseline titres between 50 and 150 RU/mL and 8/28 (29%) with baseline tires >150 RU/mL achieved clinical complete or partial remission at 18 months. A lower proportion of patients with anti-PLA2Rab ≥150 U/mL at baseline achieved immunological remission at 6 and 12 months after rituximab treatment (Supplementary data, S3b).

### Quality of life, hospitalizations and death

HRQoL EQ-5D-5L scores did not differ following rituximab between patients in either complete or partial remission and those who did not achieve remission (Supplementary data, S4).

Over the first and second rituximab doses totalling 358 administrations, there were 24 minor adverse reactions comprising urticaria, pruritus, headache, fever, nausea and hypotension (Supplementary data, S5). Two patients did not receive a second dose, due to angioedema and pruritus. Hospitalizations longer than 24 h recorded in HES were used as proxies for serious adverse events (SAEs). There were 204 hospitalizations affecting 61 people, and 11 deaths (Table 2). Approximately a third (23/61) had only one admission; one patient had 17 separate hospital admissions. There was no difference in hospitalizations as a function of remission status at 24 months [Χ^2^ (1, *N* = 105) = 1.23, *P *= .78]. Twenty-three people with infection were admitted on 37 occasions, the majority of which were due to coronavirus disease 2019 (COVID-19) and pneumonia (Supplementary data, S6).

**Table 2:** Causes of hospitalization and death during follow-up.

ICD-10 diagnosis	Number of patients affected	Number of hospitalizations^a^	Deaths^b^
Renal	30	56	1
Respiratory including lower respiratory infection	11	34	1
Circulatory	11	28	2
Metabolic	7	11	
COVID-19	5	14	3
Gastroenterological	7	10	
Abnormal clinical and lab findings	8	9	
Dermatological	6	6	1
Cancer	3	6	3
External injury or poisoning or surgical complications	4	6	
Injuries	3	6	
Neurological	3	6	
Haematological	4	4	
Infectious and parasitic disease	3	4	
Musculoskeletal	3	4	
Prior immunosuppression, *n* (%)			
Yes (*n* = 138)	49 (35)	157	10 (7)
No (*n* = 41)	14 (34)	51	1 (2)

a Total number of patients hospitalized = 61.

b Total number of deaths = 11.

Eleven people died—three from COVID-19, three from cancer, one from cellulitis, one from a pyothorax, one from gangrene, one from stroke and one from kidney failure. Baseline kidney function was low [median eGFR 21.64 (IQR 18.97–30.55)] in the 11 people who developed ESKD, 3 of whom died (Supplementary data, S8). Only increasing age was associated with a higher risk of death (HR 1.07, 95% CI 1.014–1.14). There was no significant association between baseline eGFR and associated mortality (Supplementary material, S9). There was no difference in the number of deaths as a function of previous immunosuppression [Χ^2^ (1, *N* = 179) = 1.27, *P *= .26]. It is possible that cumulative immunosuppression can influence mortality, but we were unable to show a significant difference because of the limited number of events in the two groups.

## DISCUSSION

This study represents one of the largest published experiences of rituximab use in active MN. Rituximab significantly reduced the rate of kidney function decline and induced a combined partial or complete remission rate of 54% and 60% at 18 and 24 months, respectively. The finding that rituximab reduces eGFR decline from 13.9 to 1.7 mL/min/1.73 m^2^/year is of major clinical importance given the severity of disease in this cohort. The impact of rituximab on kidney function is clinically highly significant given that 40%–50% of patients with ongoing nephrotic syndrome progress to ESKD at 10 years [18]. Furthermore, whilst MN is often perceived as a slowly progressive disease, registry data shows that ongoing nephrotic syndrome is associated with a 2- to 4-fold increase in mortality compared with the age- and sex-matched general population [19].

The baseline kidney function in our cohort was significantly worse than those reported in the four published RCTs of rituximab in MN [9, 12, 13, 15] and 19% of our cohort had an eGFR of <30 mL/min/1.73 m^2^. In contrast, the largest published trial of rituximab excluded patients with a creatinine clearance of <40 mL/min/1.73 m^2^ [13]. Eleven patients in the CtE scheme reached ESKD whereas no patients in the published RCTs of rituximab reached ESKD, which is consistent with the observation that poor baseline kidney function is strongly associated with progressive kidney failure [20]. Furthermore, 138 patients (77%) in our cohort had active disease despite previous immunosuppression. In contrast, only a total of 20 patients across three RCTs had received immunosuppression prior to rituximab [12, 13, 16]. This phenotypic difference in disease severity between the CtE cohort and patients in the trial populations, as well as data attrition in this real-world study, likely account for the slightly lower remission rates in the current cohort compared with those in previous trials [9, 12, 13, 15]. In Ruggenenti *et al*.’s study of 100 consecutive patients with rituximab and persistent nephrotic syndrome, 32 of whom had received previous immunosuppressive treatment, rates of complete or partial remission after rituximab were similar between patients with or without previous administration of immunosuppressive medications [21]. Our cohort all had absolute or relative contraindications to CNIs or cytotoxic therapy. Therefore, without rituximab most patients would have been expected to progress to ESKD within around 5 years given the presence of active disease and the trajectory of rapid eGFR decline prior to rituximab [2].

No disease characteristics at baseline could meaningfully predict remission after rituximab, although diabetes and male sex predicted longer time to remission. This is consistent with a recent observation that rituximab induces remission more quickly in women, although the mechanism behind this phenomenon is unclear [22]. Our observation that diabetes and baseline proteinuria are associated with a higher rate of decline in kidney function is consistent with findings from other cohorts [23, 24]. Of note, baseline eGFR did not predict either remission rates or impact of rituximab on kidney function decline. Whilst high anti-PLA2Rab titres are associated with more severe disease [22], we did not find that baseline anti-PLA2Rab titres predicted response to therapy or decline in kidney function in multivariate models. This suggests that rituximab can be efficacious even in high-risk MN patients. The remission rates at 12 months vs 18–24 months were different between the low compared with medium- or high-titre groups. We were unable to extrapolate the significance of these findings but believe that larger scale studies are needed to unravel this association definitively. Rituximab also reduced antibody levels within the first 3–6 months in some patients and clinical remission was more commonly reached in patients who achieved immunological remission as previously described [12, 14, 25]. Anti-PLA2Rab titres were not available in 23 patients prior to enrolment in the study. Some patients had relatively recent diagnosis whilst others had disease over much longer duration, however this did not influence remission after rituximab.

The efficacy of rituximab may depend on the dosing strategy [26]. In our cohort, 34 patients received three or more infusions, and treatment doses varying between 1 and 4 g have been used in RCTs but the optimum dosing strategy in MN is not known [9, 12, 13, 15]. Urinary loss of rituximab may be greater in patients with heavier proteinuria and therefore the depth of B-cell depletion with rituximab may be attenuated, necessitating the need for re-dosing [27]. Measuring serum rituximab levels or B-cell depletion may help tailor the dosing of rituximab [28, 29].

Patients with nephrotic syndrome have a significant symptom burden [8] but we did not find that those who achieved complete or partial remission with rituximab had significantly better HRQoL scores. This may reflect the insensitivity of the EQ-5D-5L, and patient-reported outcome measures specifically for nephrotic syndrome [30] may be a superior measure of HRQoL.

Rituximab was well tolerated with few infusion-related reactions other than angioedema and pruritus occurring in two patients. As there is no control group, it is impossible to assess how many deaths or serious adverse events are attributable to rituximab or underlying disease. Sixty-two percent of patients at baseline had an eGFR of <60 mL/min/1.73 m^2^ and patients with more severe stages of chronic kidney disease are at higher risk of hospitalization and infection [31]. Whilst the six deaths from infection could potentially be related to rituximab, uncontrolled nephrotic syndrome itself is associated with a high risk of infection and mortality [18]. Indeed, there was no difference in the frequency of reported SAEs or infections in trials comparing rituximab versus both non-immunosuppressive therapy [12] and immunosuppression with ciclosporin or cyclophosphamide [13, 14]. Three patients died from COVID-19 and rituximab both increases the risk of severe COVID-19 and reduces vaccine efficacy [32]. This suggests the need to carefully consider the risks and benefits of rituximab in elderly patients with active disease in MN and the timing of administration in relation to COVID-19 vaccination. We speculate that baseline factors, including those with advanced renal function, could have contributed to higher incidence of deaths and ESKD in this study compared with prior published studies and RCTs. The study of 100 consecutive patients [21] with rituximab in MN and nephrotic syndrome noted that four patients died and four patients reached ESKD over a median follow-up of 29 months. It is likely that the decline in eGFR leading to ESKD is related to combination of active immunological disease as well as chronic changes related to interstitial fibrosis and tubular atrophy, glomerulosclerosis, and vascular disease etc. It is still possible to achieve remission in some patients with eGFR <30 mL/min/1.73 m^2^ at or prior to rituximab, which is in keeping with other studies of patients with advanced chronic kidney disease [33, 34].

There are several limitations to this study. As patients had contraindications to CNIs and cytotoxic therapy, there was no control group; it is therefore impossible to attribute with complete certainty the observed benefits or harms of rituximab in this population. However, the design of the study with pre- and post-treatment comparison allowed for the evaluation of the effect of rituximab. It is highly unlikely that the beneficial effect of rituximab could have happened spontaneously without such treatment given the tight temporal relationship between rituximab administration and change in eGFR decline and proteinuria. Seventy-seven percent of the cohort had active disease despite receiving prior immunosuppression, suggesting that spontaneous remission without rituximab would be extremely unlikely, although the study cannot delineate the legacy effects of historical immunosuppression particularly alkylating agents. A further limitation was that patients with a very low eGFR were not excluded from this study. This was to try to capture the real-world effects of rituximab in a broad population. However, this may have resulted in patients with advanced irreversible kidney disease inappropriately receiving immunosuppression. Comprehensive blood pressure follow-up data were not available so its potential effect on proteinuria cannot be discounted, although blood pressure was reasonably well controlled at baseline. We were unable to report or discuss the historical biopsy details or historical CNI levels, which may provide more insights especially in those with declining kidney function. It was not possible to retrieve patients’ remission or relapse status when they were given additional doses of rituximab, which precludes systematic evaluation of the effect of more than two doses. As data were collected during routine clinical care there were some missing biochemical results at certain timepoints, and some patients had relatively recent diagnosis whilst others had disease over much longer duration. Despite these limitations, the study adds to our understanding of disease and use of rituximab in treatment of MN. The inclusive nature of the study meant that 180 patients were enrolled in just under 24 months, enabling the rapid generation of evidence. As the study terminated in November 2021, it was not possible to get 2-year follow-up data in all participants. However, the study has several strengths including the use of data linkage, which systematically and cost-effectively captured efficacy and safety data in a large patient sample. A critical strength is its ecological validity, capturing the real-world outcomes of patients who largely would not otherwise be represented in clinical trials. Given the expense of RCTs and concerns about their lack of generalizability to everyday practice [35], real-world data collection is an invaluable tool in complementing the evidence base derived from RCTs and can help commission new therapies for a broad patient population. Lastly, the study highlights need to explore reasons for failure of remission after standard dosing of rituximab and to evaluate further strategies in improving remission rates in MN.

This study shows that rituximab can provide significant clinical benefit to patients with active MN and as a result NHSE have now commissioned rituximab as a treatment option for MN.

## Supplementary Material

sfae179_Supplemental_File

## Data Availability

The data underlying this article will be shared on reasonable request to the corresponding author.

## References

[1] McGrogan A, Franssen CF, de Vries CS. The incidence of primary glomerulonephritis worldwide: a systematic review of the literature. Nephrol Dial Transplant 2011;26:414–30. 10.1093/ndt/gfq665 21068142

[2] Ronco P, Beck L, Debiec H et al. Membranous nephropathy. Nat Rev Dis Primers 2021;7:69. 10.1038/s41572-021-00303-z 34593809

[3] Beck LH Jr, Bonegio RG, Lambeau G et al. M-type phospholipase A2 receptor as target antigen in idiopathic membranous nephropathy. N Engl J Med 2009;361:11–21. 10.1056/NEJMoa0810457 19571279 PMC2762083

[4] De Vriese AS, Glassock RJ, Nath KA et al. A proposal for a serology-based approach to membranous nephropathy. J Am Soc Nephrol 2017;28:421–30. 10.1681/ASN.2016070776 27777266 PMC5280030

[5] Rovin BH, Adler SG, Barratt J et al. Executive summary of the KDIGO 2021 Guideline for the Management of Glomerular Diseases. Kidney Int 2021;100:753–79. 10.1016/j.kint.2021.05.015 34556300

[6] Cattran DC, Appel GB, Hebert LA et al. Cyclosporine in patients with steroid-resistant membranous nephropathy: a randomized trial. Kidney Int 2001;59:1484–90. 10.1046/j.1523-1755.2001.0590041484.x 11260412

[7] Ramachandran R, Kumar V, Bharati J et al. Long-term follow-up of cyclical cyclophosphamide and steroids versus tacrolimus and steroids in primary membranous nephropathy. Kidney Int Rep 2021;6:2653–60. 10.1016/j.ekir.2021.07.028 34622104 PMC8484506

[8] Howman A, Chapman TL, Langdon MM et al. Immunosuppression for progressive membranous nephropathy: a UK randomised controlled trial. Lancet 2013;381:744–51. 10.1016/S0140-6736(12)61566-9 23312808 PMC3590447

[9] Fernandez-Juarez G, Rojas-Rivera J, Logt AV et al. The STARMEN trial indicates that alternating treatment with corticosteroids and cyclophosphamide is superior to sequential treatment with tacrolimus and rituximab in primary membranous nephropathy. Kidney Int 2021;99:986–98. 10.1016/j.kint.2020.10.014 33166580

[10] Jha V, Ganguli A, Saha TK et al. A randomized, controlled trial of steroids and cyclophosphamide in adults with nephrotic syndrome caused by idiopathic membranous nephropathy. J Am Soc Nephrol 2007;18:1899–904. 10.1681/ASN.2007020166 17494881

[11] Ponticelli C, Zucchelli P, Passerini P et al. A 10-year follow-up of a randomized study with methylprednisolone and chlorambucil in membranous nephropathy. Kidney Int 1995;48:1600–4. 10.1038/ki.1995.453 8544420

[12] Dahan K, Debiec H, Plaisier E et al. Rituximab for severe membranous nephropathy: a 6-month trial with extended follow-up. J Am Soc Nephrol 2017;28:348–58. 10.1681/ASN.2016040449 27352623 PMC5198292

[13] Fervenza FC, Appel GB, Barbour SJ et al. Rituximab or cyclosporine in the treatment of membranous nephropathy. N Engl J Med 2019;381:36–46. 10.1056/NEJMoa1814427 31269364

[14] Beck LH Jr, Fervenza FC, Beck DM et al. Rituximab-induced depletion of anti-PLA2R autoantibodies predicts response in membranous nephropathy. J Am Soc Nephrol 2011;22:1543–50. 10.1681/ASN.2010111125 21784898 PMC3148709

[15] Remuzzi G, Chiurchiu C, Abbate M et al. Rituximab for idiopathic membranous nephropathy. Lancet North Am Ed 2002;360:923–4. 10.1016/S0140-6736(02)11042-7 12354476

[16] Scolari F, Delbarba E, Santoro D et al. Rituximab or cyclophosphamide in the treatment of membranous nephropathy: the RI-CYCLO randomized trial. J Am Soc Nephrol 2021;32:972–82. 10.1681/ASN.2020071091 33649098 PMC8017548

[17] Herdman M, Gudex C, Lloyd A et al. Development and preliminary testing of the new five-level version of EQ-5D (EQ-5D-5L). Qual Life Res 2011;20:1727–36. 10.1007/s11136-011-9903-x 21479777 PMC3220807

[18] Troyanov S, Wall CA, Miller JA et al. Idiopathic membranous nephropathy: definition and relevance of a partial remission. Kidney Int 2004;66:1199–205. 10.1111/j.1523-1755.2004.00873.x 15327418

[19] Kolb A, Gallacher PJ, Campbell J et al. A national registry study of patient and renal survival in adult nephrotic syndrome. Kidney Int Rep 2021;6:449–59. 10.1016/j.ekir.2020.10.033 33615070 PMC7879209

[20] Gupta S, Connolly J, Pepper RJ et al. Membranous nephropathy: a retrospective observational study of membranous nephropathy in north east and central London. BMC Nephrol 2017;18:201. 10.1186/s12882-017-0615-5 28637442 PMC5480139

[21] Ruggenenti P, Cravedi P, Chianca A et al. Rituximab in idiopathic membranous nephropathy. J Am Soc Nephrol 2012;23:1416–25. 10.1681/ASN.2012020181 22822077 PMC3402291

[22] Perna A, Ruggiero B, Podestà MA et al. Sexual dimorphic response to rituximab treatment: a longitudinal observational study in a large cohort of patients with primary membranous nephropathy and persistent nephrotic syndrome. Front Pharmacol 2022;13:958136. 10.3389/fphar.2022.958136 36120314 PMC9479107

[23] Couser WG . Primary membranous nephropathy. Clin J Am Soc Nephrol 2017;12:983–97. 10.2215/CJN.11761116 28550082 PMC5460716

[24] Polanco N, Gutierrez E, Covarsi A et al. Spontaneous remission of nephrotic syndrome in idiopathic membranous nephropathy. J Am Soc Nephrol 2010;21:697–704. 10.1681/ASN.2009080861 20110379 PMC2844306

[25] Ruggenenti P, Debiec H, Ruggiero B et al. Anti-phospholipase A2 receptor antibody titer predicts post-rituximab outcome of membranous nephropathy. J Am Soc Nephrol 2015;26:2545–58. 10.1681/ASN.2014070640 25804280 PMC4587688

[26] Gauckler P, Shin JI, Alberici F et al. Rituximab in membranous nephropathy. Kidney Int Rep 2021;6:881–93. 10.1016/j.ekir.2020.12.035 33912740 PMC8071613

[27] Del Vecchio L, Allinovi M, Rocco P et al. Rituximab therapy for adults with nephrotic syndromes: standard schedules or B cell-targeted therapy?. J Clin Med 2021;10:5847. 10.3390/jcm10245847 34945143 PMC8709396

[28] Cravedi P, Ruggenenti P, Sghirlanzoni MC et al. Titrating rituximab to circulating B cells to optimize lymphocytolytic therapy in idiopathic membranous nephropathy. Clin J Am Soc Nephrol 2007;2:932–7. 10.2215/CJN.01180307 17702725

[29] Seitz-Polski B, Dahan K, Debiec H et al. High-dose rituximab and early remission in PLA2R1-related membranous nephropathy. Clin J Am Soc Nephrol 2019;14:1173–82. 10.2215/CJN.11791018 31340979 PMC6682825

[30] Carter SA, Lightstone L, Cattran D et al. A core outcome set for trials in glomerular disease: a report of the Standardized Outcomes in Nephrology-Glomerular Disease (SONG-GD) stakeholder workshops. Clin J Am Soc Nephrol 2022;17:53–64. 10.2215/CJN.07840621 34969698 PMC8763157

[31] Iwagami M, Caplin B, Smeeth L et al. Chronic kidney disease and cause-specific hospitalisation: a matched cohort study using primary and secondary care patient data. Br J Gen Pract 2018;68:e512–23. 10.3399/bjgp18X697973 30012811 PMC6058621

[32] Arnold J, Winthrop K, Emery P. COVID-19 vaccination and antirheumatic therapy. Rheumatology (Oxford) 2021;60:3496–502. 10.1093/rheumatology/keab223 33710296 PMC7989162

[33] Hanset N, Esteve E, Plaisier E et al. Rituximab in patients with phospholipase A2 receptor–associated membranous nephropathy and severe CKD. Kidney Int Rep 2020;5:331–8. 10.1016/j.ekir.2019.12.006 32154454 PMC7056852

[34] Ragy O, Hamilton P, Pathi A et al. Long-term safety, clinical and immunological outcomes in primary membranous nephropathy with severe renal impairment treated with cyclophosphamide and steroid-based regimen. Glomer Dis 2023;3:88–97. 10.1159/000529605 PMC10126738 37113496

[35] Hong JC . Strategies to turn real-world data into real-world knowledge. JAMA Netw Open 2021;4:e2128045. 10.1001/jamanetworkopen.2021.28045 34618043

