# Supplementary material for: Prospective study of the effect of rituximab on kidney function in membranous nephropathy
Source: Clin Kidney J. 2024 Jun 18;17(8):sfae179. doi: 10.1093/ckj/sfae179 (PMC11299108; doi:10.1093/ckj/sfae179)
Supplement: sfae179_Supplemental_File [file sfae179_supplemental_file.docx]

**Prospective study on the effect of rituximab on kidney function in membranous nephropathy**

**Supplementary Material:**

# **Table of Contents**

List of sites and investigators Page 2

Supplementary tables and figures Page 3

S1. Baseline Comorbidities Page 3

S2. Previous immunosuppression and supportive treatment Page 3

S3a. Clinical remission status by baseline anti-PLA2Rab levels Page 4

S3b: Immunological remission status by baseline anti-PLA2Rab

levels (<150 vs ≥150 RU/mL) after rituximab treatment. Page 5

S4. Health-related quality of life scores by remission status Page 6

S5. Adverse reactions during infusions of rituximab Page 6

S6. Infection-related hospitalisations following rituximab Page 6

S7. Kaplan-Meier survival plot for ESKD Page 7

S8. Baseline Anti-PLA2Rab and eGFR in patients progressing to ESKD Page 7

S9. Relationship between baseline eGFR and mortality Page 8

PROTOCOL Page 8

1. Commissioning Through Evaluation questions Page 8

2. Study design Page 9

3. Patient population Page 10

- 1. Eligibility criteria for rituximab Page 10
  2. Definitions Page 10

4. Sample Size Page 11

5. Recruiting Centres Page 12

6. Information Governance Page 12

7. Patient Consent Page 12

8. End of study definition Page 13

9. Data Collection, Management and Statistical Analysis Page 13

9.1 Data Collection Page 13

9.2 Statistical Analysis Page 14

10. Consent Form Page 15

11. Patient Information Sheet Page 16

12. STROBE statement Page 19

# **List of investigators in NHS England hospitals**

| **Hospital** | **Investigators** |
| --- | --- |
| Basildon University Hospital | Georgia Winnett / Poorva Jain |
| Heartlands Hospital | Bamidele Ajayi |
| Queen Elizabeth Hospital, Birmingham | Peter Hewins / Jennifer Pinney |
| St Luke's Hospital | John Stoves |
| Southmead Hospital | Albert Power |
| Addenbrookes Hospital | Lisa Wilcocks |
| Cumberland Infirmary | Andrew Bow |
| St Helier Hospital | Bhrigu Sood |
| Broomfield Hospital | Sumith Abeygunasekara |
| Royal Derby Hospital | Nick Selby |
| Doncaster Royal Infirmary | Shanmugakumar Chinnappa |
| Royal Devon and Exeter Hospital | Lucy Smyth |
| Gloucestershire Royal Hospital | Preetham Boddana |
| Hull Royal Infirmary | Matthew Edey |
| St James's University Hospital | Andrew Lewington / Elizabeth Garthwaite |
| Leicester General Hospital | Chee Kay Cheung |
| Aintree University Hospital | Azri Nache |
| Royal Liverpool University Hospital | Shahed Ahmed |
| Barts & The London Hospital | Neil Ashman |
| Guy's and St Thomas's Hospital | Heather Brown |
| Royal Free Hospital | Ruth Pepper |
| St George's Hospital | Joyce Popoola |
| West London Renal & Transplant Centre | Megan Griffith |
| Manchester Royal Infirmary | Durga Kanigicherla |
| James Cook University Hospital | Caroline Wroe / Neil Hoye |
| Freeman Hospital | Laura Baines |
| Norfolk & Norwich University Hospital | Jean Patrick / Ravi Varma |
| Nottingham City Hospital | Alastair Ferraro |
| Churchill Hospital | Allie Thornley / Phil Mason |
| Derriford Hospital | Kris Houlberg / Nirosha Gunatillake / Andrew Connor |
| Queen Alexandra Hospital | Robert Lewis |
| Royal Preston Hospital | Ajay Dhaygude |
| Royal Berkshire Hospital | Oliver Flossmann |
| Salford Royal Hospital | Smeeta Sinha |
| Northern General Hospital | Arif Khwaja/Veena Reddy |
| Southend University Hospital | Gowrie Balasubramaniam |
| Lister Hospital | Barbara Thompson / Enric Vilar |
| Royal Stoke University Hospital | Julie Wessels |
| Arrowe Park Hospital | Indiver Daryanani |

# **Supplementary tables and Figures**

## S1: Baseline Comorbidities

| Comorbidities | |
| --- | --- |
| Diabetes | 28 (16%) |
| Ischaemic heart disease | 13 (7%) |
| Cardiac failure | 1 (1%) |
| Lung disease | 11 (6%) |
| Peripheral vascular disease | 4 (2%) |
| CNS abnormalities | 5 (3%) |
| Liver cirrhosis | 1 (1%) |
| History of cancer | 8 (4%) |
| History of venous thromboembolism | 15 (8%) |
| Any comorbidity* | 66 (37%) |
| Data are n (%) for 180 patients.  CNS – central nervous system.  *Some people have more than one comorbidity. | |

**S2: Previous immunosuppression and supportive treatment**

| **Previous immunosuppression and supportive treatment** | |
| --- | --- |
| Total number of people on other treatments | 138 (77%) |
| Alkylating agents (in combination or alone) | 88 (49%) |
| Alkylating agents alone | 40 (22%) |
| CNIs (in combination or alone) | 84 (47%) |
| CNIs alone | 36 (20%) |
| Tacrolimus alone | 26 (14%) |
| Cyclosporin A alone | 10 (6%) |
| Steroids | 111 (62%) |
| Rituximab (originator or biosimilar) | 6 (3%) |
| Anti-proliferatives (including azathioprine and mycophenolate mofetil) | 8 (4%) |
| RAAS inhibitors | 159 (88%) |
| Statins | 132 (73%) |
| Diuretics | 128 (71%) |
| Anti-platelets | 32 (18%) |
| Anti-coagulants | 78 (43%) |
| Data are n (%) for 180 patients.  CNI – calcineurin inhibitors; RAAS – renin angiotensin aldosterone system. | |

## S3a: Clinical remission status by baseline anti-PLA2Rab levels

|  | Anti-PLA2Rab  Neg  (n=39) | Anti-PLA2Rab  14-50  (n=33) | Anti-PLA2Rab  51-150 (n=44) | Anti-PLA2Rab  >150  (n=37) | Anti-PLA2Rab  Unknown  (n=27) | Total  n=180 |
| --- | --- | --- | --- | --- | --- | --- |
| **Death** | 3 (8%) | 1 (3%) | 3 (7%) | 3 (8%) | 1 (4%) | 11 (6%) |
| **RRT** | 3 (8%) | 1 (3%) | 3 (7%) | 4 (11%) | 0 (0%) | 11 (6%) |
| **12-month PR or CR** | 15/32 (47%) | 12/25 (48%) | 17/37 (46%) | 11/29 (39%) | 11/21 (52%) | 66/143 (46%) |
| **18-month PR or CR** | 12/28 (43%) | 9/27 (33%) | 15/36 (42%) | 8/28 (29%) | 11/20 (55%) | 55/139 (40%) |
| **24-month PR or CR** | 11/23 (48%) | 4/15 (27%) | 15/30 (50%) | 9/25 (36%) | 4/12 (33%) | 43/105 (41%) |

Table S3a: shows the outcomes by baseline anti-PLA2Rab levels by death, renal replacement therapy (RRT), complete remission (CR) and partial remission (PR)

## S3b: Immunological remission status by baseline anti-PLA2Rab levels (<150 vs ≥150 RU/mL) after rituximab treatment.


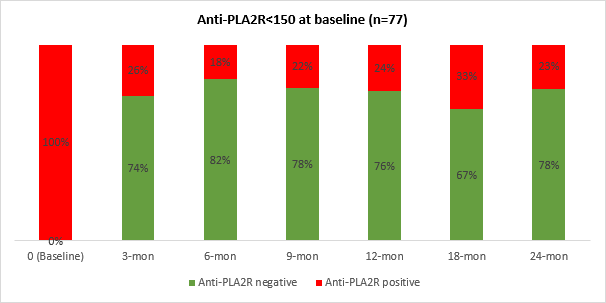


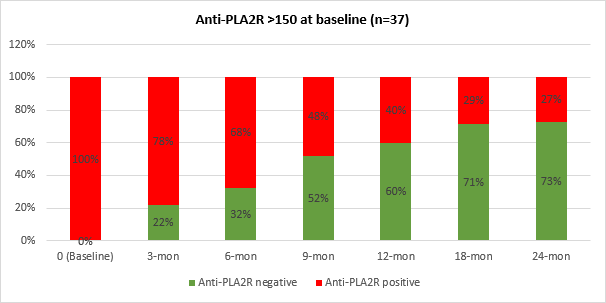


## S4: Health-related quality of life scores (EQ-5d-5L) by remission status at 12 and 24 months

| Timepoint | 12 months | 24 months |
| --- | --- | --- |
| Partial remission | 0·89 (0·17)  n=47 | 0·90 (0·11)  n=31 |
| Complete remission | 0·91 (0·08)  n=5 | 0·92 (0·10)  n=14 |
| No remission | 0·85 (0·18)  n=47 | 0·86 (0·16)  n=55 |
| Data are mean (SD) as assessed by the EQ-5D-5L. | | |

## S5: Adverse reactions during first and second infusions of rituximab

| Reaction | First dose | Second dose |
| --- | --- | --- |
| Urticaria | 6 | 1 |
| Pruritis | 4 | 0 |
| Headache | 3 | 2* |
| Fever | 2 | 1 |
| Nausea | 0 | 1 |
| Angioedema | 2 | 0 |
| Hypotension/dizziness | 1 | 1* |
| Data represent number of documented incidences.  *Recurring in the same patient. | | |

## S6: Infection-related hospitalisations following rituximab

| Type of infection | Patients affected | Number of hospitalisations |
| --- | --- | --- |
| COVID-19 | 5 | 14 |
| Pneumonia | 8 | 14 |
| Lower respiratory | 3 | 3 |
| Urinary | 1 | 1 |
| Gastroenteritis | 1 | 1 |
| Infected implant | 1 | 1 |
| Sepsis | 2 | 3 |
| Total | 21 | 37 |

## S7. Kaplan-Meier survival plot for ESKD


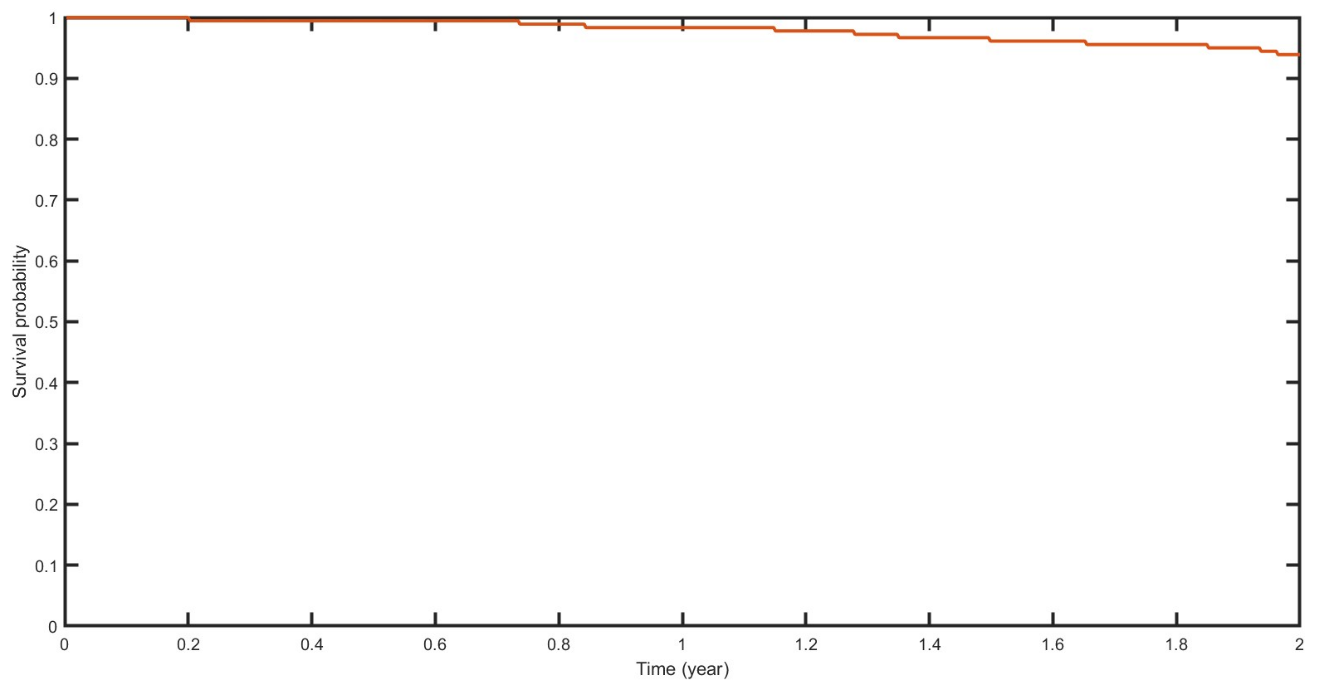


## S8. Baseline Anti-PLA2Rab and eGFR in patients progressing to ESKD

| Case | Dialysis | Transplant | Death | eGFR | AntiPLA2R |
| --- | --- | --- | --- | --- | --- |
| 3725 | Y |  |  | 21.64 | 3 |
| 5196 | Y |  |  | 19.12 | 33 |
| 10571 | Y |  |  | 21.15 | 3 |
| 23080 | Y |  |  | 90.16 | 3 |
| 23208 | Y |  |  | 18.76 | 176 |
| 23806 | Y |  | Y | 36.85 | 270 |
| 24611 | Y |  |  | 21.87 | 210 |
| 24709 | Y |  | Y | 77.11 | 67 |
| 25353 | Y |  | Y | 16.79 | 104 |
| 26400 | Y |  |  | 18.82 | 67 |
| 27741 | Y | Y |  | 24.24 | 515 |

eGFR: Q1=18.97, Q2(median)=21.64, Q3= 30.55

Anti-PLA2R: Q1=18, Q2(median)=67, Q3=193

## S9. Relationship between baseline eGFR and mortality

|  | | Death | | Total |
| --- | --- | --- | --- | --- |
|  |  | YES | NO |  |
| eGFR<30 | YES | 2 | 32 | 34 |
|  | NO | 9 | 137 | 146 |
| Total | | 11 | 169 | 180 |

Chi-square test: 0.0040

P-value: 0.95

# **PROTOCOL**

## 1. Commissioning through Evaluation (CtE) questions

This current project has been commissioned by the National Institute for Health and Care Excellence (NICE) to support NHS England (NHSE) in its Commissioning through Evaluation (CtE) programme. CtE was launched to provide funding for a limited number of patients to access medical treatments and technologies not routinely commissioned within the NHS (National Health Service England 2014). Rituximab is one such treatment which was selected for CtE. The key objective of the King’s Technology Evaluation Centre (KiTEC), a NICE external assessment centre (EAC) based at King’s College London, is to analyse clinical outcomes data related with rituximab treatment for patients participating to the CtE. This analysis will provide results regarding the clinical effectiveness of rituximab (as well as other outcomes of interest), which will assist NHS England in making its commissioning decisions. Specifically, the following evaluation questions will be addressed in an uncontrolled series of patients:

1. The primary question is how effective rituximab within patient comparison is, for the clinical indication covered within the CtE scheme in the following outcomes:
   - 1. in reducing the decline of renal function as measured by GFR
     2. in the induction of remission (partial or complete) of nephrotic syndrome
2. Does treatment with rituximab for the clinical indication covered within the CtE scheme result in fewer hospitalisations and better quality of life (QoL) in comparison with best supportive care?
   - 1. QoL will be assessed by EQ-5D (in line with the NICE Reference Case)
3. What is the relationship between the following patient characteristics and the outcomes outlined in the primary question:
   - 1. Age
     2. Baseline renal function (proteinuria, Glomerular Filtration Rate [eGFR])
     3. Sex
     4. anti-phospholipase A2 receptor antibody (PLA2R-Ab) status and PLA2R-Ab titre
     5. Prior immunosuppression
4. Do PLA2R-Ab levels correlate with disease activity (active disease, partial remission, complete remission) in patients treated with rituximab? Can anti-phospholipase A2 receptor antibody levels be used to:
   - 1. Monitor the response to treatment with rituximab originator product and its biosimilars?
     2. Predict disease relapse?
5. Does treatment with rituximab for the clinical indication covered within the CtE scheme result in a favourable toxicity profile compared with best supportive care and immunomodulatory treatment?
6. Does the use of rituximab for the clinical indications covered within the CtE scheme present different safety issues from their established uses?
7. What is the actual cost, and relative cost effectiveness, of treatment with rituximab for the clinical indication covered within the CtE scheme?
8. Are there any research findings relevant to rituximab that have become available during the course of the CtE scheme and which should be considered alongside the evaluative findings of the CtE scheme?

There are also 2 additional questions related to policy development:

1. Are there any factors identified during the provision of the scheme at the participating centres that should be taken into account if the service is commissioned in the future?
2. What is the estimated cost of full roll out per year if rituximab was routinely commissioned for the clinical indication covered within the CtE scheme?

## 2. Study Design

This is a prospective multi-centre national database (register) project to obtain the evidence related to rituximab that will address the research question detailed in section 1.2. The CtE programme is forecast to enable 180 patients a year to access rituximab treatment as part of a formal evaluation (non-routine commissioning) programme. The Steering Group for the programme agreed that prospective comparative data gathering is not feasible. Therefore, in the absence of comparator data, binary outcomes (remission) will be estimated and compared to published values. Changes in continuous outcomes post treatment such as GFR, will be assessed within patients.

## 3. Patient Population

### **3.1 Eligibility criteria for rituximab**

All patients eligible to receive rituximab and who have consented for their data to be entered onto the CtE register with RaDaR will be included in the analysis. The eligibility criteria for receiving rituximab treatment under CtE are set out below.

The patient population comprises of 2 cohorts and includes patients eligible to receive rituximab for IMN with either native or transplanted kidneys. The eligibility criteria are outlined below:

1. A proven diagnosis of Membranous Nephropathy and patient registered on RaDaR.
2. Maximum tolerated renin angiotensin aldosterone system (RAAS) blockade
3. Exclusion of secondary causes
4. Ongoing severe disease
5. Failure or intolerance of conventional immunomodulatory therapies
   1. For patients with eGFR greater than or equal to 60ml/min/1.73m2 this should be failure/intolerance of both an alkylating agent and a calcineurin inhibitor (CNI).
   2. For patients with and eGFR of less than 60ml/min/1.73m2 only failure/intolerance to alkylating agents is a requirement.
   3. Patients with threatened fertility who have experienced failure/intolerance of CNI.
   4. Recurrent MN after kidney transplantation (these patients are already on CNI)
6. Measurement of anti-PLA2R prior to rituximab therapy, quarterly in the first year and every 6 months thereafter

### **3.2 Definitions**

Ongoing severe disease is defined as either:

- 1. Proteinuria of greater than 3.5g/day (or Protein Creatinine Ratio [PCR] of greater than 350mg/mmol) and a serum albumin of less than 30g/l
  2. Fall in eGFR of at least 20% over at least 3 months or longer, based on a minimum of three measurements

Failure of therapy is defined as:

- 1. Ongoing severe disease (as defined above) at least 3 months after completion of alkylating agent therapy or CNI therapy

Intolerance is defined as:

- 1. Type 1 hypersensitivity reactions
  2. For alkylating agents:
     1. Bone marrow suppression, hepatitis or other drug associated toxicity greater than Grade 3 by Common Terminology Criteria for Adverse Events (CTCAE).
     2. Previous hospitalisation with infectious complication of alkylating agent therapy.
     3. Inability to comply with monitoring requirements.
     4. Previous urothelial cancer or any current cancer (excluding squamous cell carcinoma of the skin).
     5. People with threatened fertility
  3. For CNIs:
     1. Fall in eGFR on CNI therapy of at least 20% or to below 60 ml/min/1.73m2
     2. Previous hospitalisation with infectious or metabolic complication of CNI therapy
     3. Inability to comply with monitoring requirements.
  4. For any therapy that utilises adjunctive steroids (i.e., in combination with cytotoxic therapy or calcineurin inhibitors):
     1. a) Diabetes
     2. b) Risk factors for steroid induced diabetes: BMI greater than or equal to 30kg/m2 (or greater than or equal to 25kg/m2 if South Asian), family history of diabetes affecting at least one first-degree relative)

Complete response (CR) is defined as resolution of proteinuria to less than 0.30/day or a PCR of less than 30mg/mmol.

Partial response (PR) is defined as improvement in proteinuria to less than 3.5g/day or PCR less than 350mg/mmol and a fall from baseline value of at least 50%.

**4. Sample Size**

The number of patients receiving rituximab in England as part of the CtE programme is fixed and dependent on the funding available from NHSE. No formal sample size calculations were performed. NHSE has estimated that 90-180 patients will be eligible for the CtE programme based on the following:

1. Assumes a midpoint incidence of 8pmp for MN of which 6pmp is idiopathic of which 67% are progressive.
2. A refractory to conventional treatment rate in progressive cases is estimated at 15-30% based on published RCTs.

It is possible that this is an over-estimate and an incidence of 100 new cases per annum would yield 10-20 cases meeting the criteria. In addition, there are already 1030 prevalent patients on RaDaR and a proportion of these will meet the criteria.

**5. Recruiting Centres**

A total of 52 sites have been selected by NHSE to provide rituximab treatments for patients participating in the CtE. Patient recruitment to the CtE programme will be kept under regular review by the Steering group to address in time any issues with poor recruitment.

**6. Information Governance**

The CtE rituximab treatment protocol is not a research project, it is a national evaluation study of a treatment that required clinical follow-up and information gathered locally by the treating team as per normal practice. NHS England is not able to fund rituximab for IMN unless patients are registered within the CtE program. The evaluation carried out by KITEC, to analyse the data collected in the service evaluation, is effectively a registry data analysis. KiTEC’s role in the CtE project is to manage the data collection and undertake all analyses and reporting of results. In order for patients to have their data analysed by a 3rd party, their consent must be obtained. This will be covered by the RaDaR consent form which all patients taking part in the registry must sign. KiTEC will submit an application for global REC approval for analysing these patient data and will set up a data sharing agreement with the RaDaR registry. The registry already has appropriate ethical approval and consenting procedures to cover data collection for the CtE evaluation. KiTEC is applying for Research Ethics Committee (REC) approval to analyse the data only.

**7. Patient Consent**

All patients with IMN who agree to have their data uploaded to the RaDaR registry have to sign the registry’s consent form. The consent form (see appendix 1) allows for the patients’ past, present and future clinical data to be used for ongoing and future research into kidney disease and related conditions. This includes linking the RaDaR records to data obtained from the GP and hospital records. These include NHS Digital for the Hospital Episode Statistics and Office for National Statistics databases, Health Education England, the UK Renal Registry and any UK-based bio-banking scheme. NHSE will also circulate to the centres a Patient information leaflet explaining the purpose of the CtE (please see appendix 2).

**8. End of study definition**

The CtE is a 3-year programme that generated data for uploading to the RaDaR registry until June 2021. Due to the impact of Covid-19 this deadline was extended to November 2021

**9. Data Collection, Management and Statistical Analysis**

**9.1 Data Collection**

Centres delivering rituximab for the CtE project are required to record the following data for analysis at the end of the evaluation period:

- Retrospective laboratory and clinical data from the point of first diagnosis of IMN, as defined by clinical judgment
- Baseline clinical assessment and demographics, including prior treatment history up to the date of diagnosis with IMN
- Details of rituximab treatment
- Follow-up clinical assessment and response to treatment
- Acute toxicity and late toxicity both using the Common Terminology Criteria for Adverse Events (CTCAE) v 4.0
- Quality of life, using the EuroQol EQ-5D questionnaire

KiTEC has developed an agreed dataset outlining in detail the data collected as part of the CtE project (appendix 3). These data will be collected at baseline and at predefined follow-up time points as outlined in Table 1 below. Follow up beyond 2 years should be as per routine practice. These data will add to the current evidence base for the rituximab indications to be included in this evaluation and help refine patient selection criteria if rituximab was commissioned in the future.

**Table 1: Outline follow-up protocol for all sites**

| **Forms** | **Retrospective** | **Baseline** | **3 Months** | **6 Months** | **9 Months** | **12 Months** | **18 Months** | **24 Months** |
| --- | --- | --- | --- | --- | --- | --- | --- | --- |
| Demographics |  | ✓ |  |  |  |  |  |  |
| Clinical assessment retrospective (at diagnosis) | ✓ |  |  |  |  |  |  |  |
| Clinical Assessment - Baseline |  | √ |  |  |  |  |  |  |
| Rituximab administration |  | ✓ | ✓ | ✓ | ✓ | ✓ | ✓ | ✓ |
| Clinical Assessment - Follow Up |  |  | ✓ | ✓ | ✓ | ✓ | ✓ | ✓ |
| EQ-5D |  | ✓ |  |  |  | ✓ |  | ✓ |
| CTCAE |  | ✓ | ✓ | ✓ | ✓ | ✓ | ✓ | ✓ |
| Death |  |  | ✓ | ✓ | ✓ | ✓ | ✓ | ✓ |
| Re-treatment |  |  | ✓ | ✓ | ✓ | ✓ | ✓ | ✓ |

**9.2 Statistical analysis**

The statistical analysis addresses the agreed NHSE evaluation questions. Descriptive statistics will be presented to characterise the patient populations. This will include demographic and clinical factors. The proportions of patients with complete and partial remission at 12, 18 and 24 months will be presented as well as rate of change of eGFR slope pre and post-rituximab.

**Consent** form

**Patient Information Sheet**


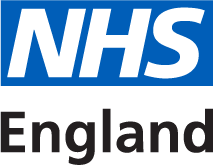


**Participant Information Sheet (PIS)**

**Programme title:** Commissioning through Evaluation (CtE) on the use of rituximab for Idiopathic Membranous Nephropathy (IMN)

**What does this information sheet contain?**

This information sheet will explain the background to rituximab treatment and provide information on the NHS England commissioning through evaluation programme.

**What is the purpose of this study?**

Some patients with idiopathic membranous nephropathy are not suitable or do not respond to conventional treatment. However, if you are not suitable for these drugs, or the drugs don’t work or the disease becomes more active despite treatment, then your doctor may consider giving you rituximab. At present, rituximab only has a license for use in treating a type of blood cancer and other auto-immune diseases. However, there is some emerging evidence showing that there may be a benefit in the use of rituximab for the treatment of idiopathic membranous nephropathy.

As part of this study, patients with idiopathic membranous nephropathy receiving rituximab treatment will be asked to provide their quality of life information before and after treatment. Clinical information will be collected at the same time. This information will allow us to expand the current evidence base and, depending on the results, routinely fund rituximab for patients with idiopathic membranous nephropathy on the NHS. The information collected about you as part of this evaluation will also help identify future research questions and studies.

**What is rituximab?**

Rituximab is a medicine used to reduce the activity of the immune system when it is overactive. It contains a protein called a ’monoclonal antibody’ which sticks to the surface of a specific type of white blood cell called a ’B-cell’. B cells are involved in making the antibodies responsible for the symptoms of IMN. When rituximab sticks to the surface of this cell, the cell dies.

**Why have I been invited?**

You have been invited because you are eligible for treatment with rituximab for your idiopathic membranous nephropathy disease.

**Do I have to take part?**

No. It is up to you to decide whether you wish to participate in the commissioning through evaluation programme. However, NHS England is not able to fund rituximab for idiopathic membranous nephropathy unless patients are registered within the commissioning through evaluation programme.

**What is the Commissioning through Evaluation (CtE) programme?**

The aim of the commissioning through evaluation programme is to gather more data on the effectiveness of the treatment, which is not currently routinely funded by the NHS, but nonetheless may show significant promise as a future treatment. It is provided by NHS England, the body responsible for delivering new treatments in the NHS.

Rituximab for idiopathic membranous nephropathy is a relatively new treatment. Current research indicates that rituximab has potential clinical advantages and relatively few side effects. However, there are few published clinical trials comparing rituximab with established treatments. NHS England has provided funding for a limited time to allow assessment of whether rituximab is beneficial to patients.

For this reason, it is an essential part of the commissioning through evaluation process that the clinical team involved with your care collects information on any side-effects of this treatment you may experience. This will be done using questionnaires. It is also vital that information is collected on how your disease responds to treatment in the future (for example, checking/having blood tests). This information will be used to help NHS England to decide whether to make rituximab for idiopathic membranous nephropathy routinely available for NHS patients.

As part of this treatment, you and your doctor should agree that this information can be collected for a period of 2 years after your treatment with rituximab. It is the responsibility of the hospital where you are treated with rituximab to collect this data.

**What data will be collected and how will they be analysed?**

This evaluation aims to collect information about clinical outcomes in patients who are treated using rituximab for comparison with historical data for similar populations.

As part of this evaluation the centres delivering rituximab treatment will be collecting data about whether you experience any adverse events because of your rituximab treatment. The study would also benefit from information about your experience of treatment with rituximab and its impact on your quality of life which will be collected using questionnaires. More specifically the following information will be collected as part of the rituximab commissioning through evaluation project:

1. Clinical assessment at the time you were diagnosed with idiopathic membranous nephropathy prior to start your treatment with rituximab, at start of treatment with rituximab and at follow-up (every 3 months for the 1 year and every 6 months for the 2-year post treatment).
2. Treatment toxicity
3. Quality of life

Taking part in the scheme will not entail extra visits to the hospital but simply involves agreeing to share information about your kidney function, any side affects you have had with treatment and filling in some brief questionnaires about your quality of life.

**Will my taking part be kept confidential?**

Yes. Ethical and legal practice will be followed and all information about you will be handled in confidence.

Identifiable data about your treatment will be collected and entered into the National Renal Rare Disease registry (RaDaR) by a member of your hospital’s [name of the NHS Trust patient is having rituximab treatment] research team. Your information will be available to you online via a website called Patient View if your hospital is signed up to this. RaDaR can only be accessed within the NHS by the named clinicians and individuals at RaDaR involved in the project. The lead Clinician for idiopathic membranous nephropathy commissioning through evaluation project will be able to see the anonymised data of all patients but no other clinicians from other Trusts. As part of this study, you would also need to consent to RaDaR and Patient View. All access to your data will be managed whilst adhering to strict protocols. Once the data is collected it will be sent in non-identifiable form to the King’s Technology Evaluation Centre (KiTEC) at King’s College, London for analysis.

At KiTEC, all of your information will be stored on password protected computers and will only be accessible to the named individuals on the team who will be carrying out analysis of your data. When the results of the evaluation are reported, individuals who have taken part will not be identified in any way. Responsible members of King’s College London may be given access to data for monitoring or audit of the evaluation to ensure we are complying with regulations.

At completion of the commissioning through evaluation programme, data from the registry will be kept for no less than 5 years and at least 2 years post-publication for any further review and analysis that may be required. The exact period of data retention after the database completion will be stipulated by NHS England and NICE who will use it to make further decisions on how this treatment could be used in the future.

**Additional information**

After your rituximab treatment you will continue to be followed up by your nephrology team as part of your standard clinical care.

If you are unsure about anything, please discuss it with the team treating you. You can also call the hospital once your treatment is finished if you need advice.

There are no expenses or payments available for participating in this study.

**What if there is a problem?**

If you have a concern about any aspect of this evaluation, you should ask to speak to your doctor who will do their best to answer your questions.

**STROBE Statement**

Checklist of items that should be included in reports of ***cohort studies****.*

|  | Item No | Recommendation | Page No |
| --- | --- | --- | --- |
| **Title and abstract** | 1 | (*a*) Indicate the study’s design with a commonly used term in the title or the abstract | 2 |
|  |  | (*b*) Provide in the abstract an informative and balanced summary of what was done and what was found | 3 |
| Introduction | | | |
| Background/rationale | 2 | Explain the scientific background and rationale for the investigation being reported | 5-6 |
| Objectives | 3 | State specific objectives, including any prespecified hypotheses | 6 |
| Methods | | | |
| Study design | 4 | Present key elements of study design early in the paper | 7 |
| Setting | 5 | Describe the setting, locations, and relevant dates, including periods of recruitment, exposure, follow-up, and data collection | 7-8 |
| Participants | 6 | (*a*) Give the eligibility criteria, and the sources and methods of selection of participants. Describe methods of follow-up | 7-8 |
|  |  | (*b*) For matched studies, give matching criteria and number of exposed and unexposed |  |
| Variables | 7 | Clearly define all outcomes, exposures, predictors, potential confounders, and effect modifiers. Give diagnostic criteria, if applicable | 8-9 |
| Data sources/ measurement | 8* | For each variable of interest, give sources of data and details of methods of assessment (measurement). Describe comparability of assessment methods if there is more than one group | 8-9 |
| Bias | 9 | Describe any efforts to address potential sources of bias | 8 |
| Study size | 10 | Explain how the study size was arrived at | 8 |
| Quantitative variables | 11 | Explain how quantitative variables were handled in the analyses. If applicable, describe which groupings were chosen and why | 8-9 |
| Statistical methods | 12 | (*a*) Describe all statistical methods, including those used to control for confounding | 8-9 |
|  |  | (*b*) Describe any methods used to examine subgroups and interactions | 8-9 |
|  |  | (*c*) Explain how missing data were addressed | 8-9 |
|  |  | (*d*) If applicable, explain how loss to follow-up was addressed | 8-9 |
|  |  | (*e*) Describe any sensitivity analyses | 8-9 |
| Results | | |  |
| Participants | 13* | (a) Report numbers of individuals at each stage of study—eg numbers potentially eligible, examined for eligibility, confirmed eligible, included in the study, completing follow-up, and analysed | 10 |
|  |  | (b) Give reasons for non-participation at each stage | 10 |
|  |  | (c) Consider use of a flow diagram |  |
| Descriptive data | 14* | (a) Give characteristics of study participants (eg demographic, clinical, social) and information on exposures and potential confounders | 10,24 |
|  |  | (b) Indicate number of participants with missing data for each variable of interest | 24-26 |
|  |  | (c) Summarise follow-up time (eg, average and total amount) | 10-12, 24-26 |
| Outcome data | 15* | Report numbers of outcome events or summary measures over time | 10-12,24,26 |

| Main results | 16 | (*a*) Give unadjusted estimates and, if applicable, confounder-adjusted estimates and their precision (eg, 95% confidence interval). Make clear which confounders were adjusted for and why they were included | 10-11,24-26 |  |
| --- | --- | --- | --- | --- |
|  |  | (*b*) Report category boundaries when continuous variables were categorized | 1-12,24-26 |  |
|  |  | (*c*) If relevant, consider translating estimates of relative risk into absolute risk for a meaningful time period |  |  |
| Other analyses | 17 | Report other analyses done—eg analyses of subgroups and interactions, and sensitivity analyses | 10-12 |  |
| Discussion | | | | |
| Key results | 18 | Summarise key results with reference to study objectives | 14-15 |  |
| Limitations | 19 | Discuss limitations of the study, taking into account sources of potential bias or imprecision. Discuss both direction and magnitude of any potential bias | 16 |  |
| Interpretation | 20 | Give a cautious overall interpretation of results considering objectives, limitations, multiplicity of analyses, results from similar studies, and other relevant evidence | 14-16 |  |
| Generalisability | 21 | Discuss the generalisability (external validity) of the study results | 17 |  |
| Other information | | | | |
| Funding | 22 | Give the source of funding and the role of the funders for the present study and, if applicable, for the original study on which the present article is based | 2 |  |

*Give information separately for exposed and unexposed groups.
